# Whole genome sequencing identifies a novel *ALMS1* gene mutation in two Chinese siblings with Alström syndrome

**DOI:** 10.1186/s12881-017-0418-3

**Published:** 2017-07-19

**Authors:** Lin Yang, Zixiu Li, Mei Mei, Xiaomei Fan, Guodong Zhan, Huijun Wang, Guoying Huang, Mingbang Wang, Weidong Tian, Wenhao Zhou

**Affiliations:** 1 Division of Endocrinology, Genetics and Metabolic Diseases, Children’s Hospital of Fudan University, Shanghai, China; 2 Key Laboratory of Birth Defects, Children’s Hospital of Fudan University, Shanghai, China; 3 Department of Biostatistics and Computational Biology, Life Science, Fudan University, Shanghai, China; 4 Division of Respiration, Children’s Hospital of Fudan University, Shanghai, China; 5 BGI technology, Shanghai, China; 6 Key Laboratory of Neonatal Diseases, Ministry of Health, Children’s Hospital of Fudan University, Shanghai, China; 7 Department of Neonates, Children’s Hospital, Fudan University, 399 Wan Yuan Road, Shanghai, China 201102

**Keywords:** Alström syndrome, Whole genome sequencing, *ALMS1* gene, Cone-rod dystrophy

## Abstract

**Background:**

Alström syndrome is a rare multi-systemic disorder with a broad spectrum of symptoms. This syndrome is characterized by childhood retinal degeneration; sensorineural hearing loss; obesity; type 2 diabetes mellitus; cardiomyopathy; systemic fibrosis; and pulmonary, hepatic, and renal failure.

**Case presentation:**

A Chinese quartet family with two siblings predominantly affected by cone-rod dystrophy and short stature were recruited. The craniofacial dysmorphism and on-set age-of-cone-rod dystrophy in the proband showed a minor intrafamilial variability. Whole genome sequencing was performed to provide the full spectrum of the two siblings’ genetic variations. In this study, we present the patients’ clinical features and our interpretation of the whole genome sequencing data. After examining the data, we focus on two compound heterozygous mutations, (c.3902C > A, p.S1301X; c.6436C > T, p.R2146X) in *ALMS1,* which are shared by two siblings.

**Conclusion:**

We reported a novel *ALMS1* mutation. Whole genome sequencing is a powerful tool to provide the full spectrum of genetic variations for heterogeneous disorders such as Alström syndrome.

**Electronic supplementary material:**

The online version of this article (doi:10.1186/s12881-017-0418-3) contains supplementary material, which is available to authorized users.

## Background

Alström syndrome (ALMS; MIM# 203800) is a rare multi-systemic autosomal recessive disorder with a broad spectrum of clinical symptoms, including progressive cone-rod dystrophy, sensorineural hearing impairment, truncal obesity, insulin resistance, hypogonadism, dilated or restrictive cardiomyopathy, and multiple organ failure [1, 2]. Alström syndrome is caused by mutations in the ALMS1 gene.

In the past 3 years, several new causative genes for at least 150 heritable disorders, and mutations in known genes for unexplained phenotypes, have been discovered by using the whole-exome sequencing (WES) or whole-genome sequencing (WGS) techniques [3]. Protein-coding genes comprise only approximately 1% of the human genome but harbor 85% of the mutations with substantial effects on disease-related traits [4], which makes WES a cost-effective method for detecting coding-region mutations. Nevertheless, WES involves a hybridization step by which the coding regions are captured from the whole genomic DNA, and during this process, 3 to 5% of the targets will be missed. Furthermore, the full spectrum of copy number variations (CNVs) and breakpoints may not be completely characterized by WES [5]. In contrast to WES, WGS avoids these limitations, and its quartet framework offers an analytical advantage for the detection and correction of sequencing errors [6]. As the price of generating whole-exome and whole-genome data continues to drop and the price difference between WES and WGS continues to narrow, WGS is anticipated to become more widely used in clinical settings [7]. A Chinese quartet family with two siblings primarily affected by cone-rod dystrophy were recruited. To provide for the full spectrum of the patients’ genetic variations, whole-genome sequencing (WGS) was performed to search for candidate point mutations, InDels and CNVs. In this study, we presented the patients’ clinical features and our interpretation of the WGS data. We finally focus on two compound heterozygous mutations in *ALMS1* shared by two siblings.

## Case presentation

### Clinical studies

At the time of admission, the proband was a 16-year-old boy with a height of 155 cm (<3^rd^ centile) (short stature, HP:0004322) and weight of 57 kg (25^th^–50^th^ percentile) [8]. He had craniofacial dysmorphism (round face HP:0000311, frontal balding HP: 0002292, thin hair HP: 0002213, narrow palpebral fissures HP: 0000581, and bitemporal flatting HP: 0000272, Fig. 1b). There was no evidence of hypponadism at the age of 16 years, with normal testicular volume and normal penis length, with well-developed cavernous. He had a history of photophobia (HP: 0000613) (HP: 0000613) and nystagmus (HP: 0000639) beginning at less than 3 months of age, which eventually resulted in blindness (HP: 0000618) by the age of 7–8 years. Bilateral sensorineural hearing loss (HP: 0000408) developed at the age of 8–9 years. Diabetes (HP: 0000873) developed at the age of 14 and was treated with subcutaneous insulin injections. Obesity developed (HP: 0001513) before 15 years. The ophthalmologic examination was notable for cone-rod dystrophy (HP: 0000548). Abdominal ultrasound found multiple solid masses (5.1–10.3 mm) in the liver. Echocardiography did not indicate any abnormalities at this time. There was no evidence of developmental delays, mental retardation, cardiologic impairment, hypertension, polydactyly, or hypogonadism. Serum concentration levels of triglyceridemia and blood urea nitrogen were slightly elevated, but creatinine, aspartate aminotransferase, alanine, and aminotransferase levels were normal.

**Fig. 1:**
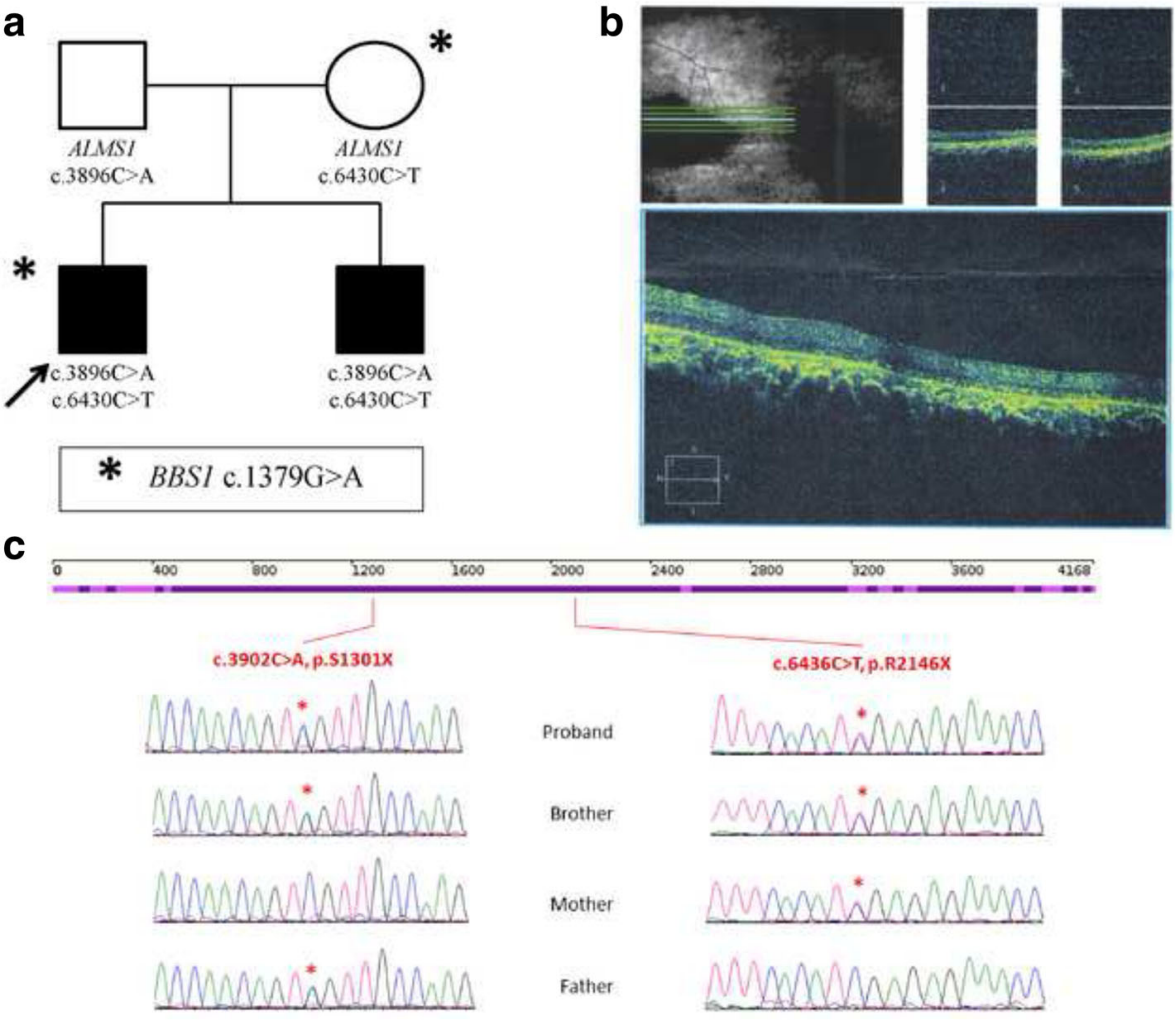
Pedigrees of the family, Sanger sequencing confirmation and Optical coherence tomography of the proband. **a** Pedigrees of the quartet families. **b** Optical coherence tomography of the proband reveals atrophy of the retina with epiretinal membrane and retina pigment epithelium irregularly arrangement. **c** Sanger sequencing confirmation in the quartet family. Heterozygous nucleotide and amino acid substitutions are shown at the top. Presence of the mutated alleles is indicated by a *red asterisk*

The younger brother is 8 years old, with a height of 120 cm (3^rd^ percentile) and weight 30 kg (50^th^–75^th^ percentile). There were no obvious malformations such as craniofacial dysmorphism or obesity (BMI = 20.83 kg/m^2^). There was no evidence of hypponadism at this time. The boy had a history of photophobia, nystagmus, and progressive cone-rod retinal dystrophy beginning at 3 months of age. Vision was 0.1 when evaluated. There was no evidence of hearing loss, diabetes, development delay, or mental retardation. Ophthalmologic examination revealed mild cone-rod dystrophy. Examining abdominal ultrasound, echocardiography, liver function and renal function did not produce evidence of any further abnormalities.

The parents of the two boys were both healthy, non-consanguineous adults. There was no family history of congenital malformations and no known teratogenic exposure. The major clinical manifestations of ALMS are shown in Table 1.

**Table 1:** Medical history and clinical features in the proband and his brother

	Features	Proband (age: 16.7 years)	Brother (age: 8 years)
Face	craniofacial dysmorphism	+	-
Cone-rod dystrophy	onset age	<3 months	>3 months
	photophobia	+	+
	nystagmus	+	+
	Blindness	7–8 years	-
	Full-field electroretinography (ERG)	+	±
	Fundus examination	+	+
Obesity	Obesity	+	-
Hearing loss	onset age	8–9 years	-
	glue ear (long-standing sticky fluid in the middle ear)	-	-
	Chronic otitis media	-	-
	Sensorineural impairment	+	-
Diabetes	onset age	14 years	-
	plasma insulin concentration	+	-
	glucose intolerance	+	-
	insulin resistance	-	-
	acanthosis nigricans	-	-
	Coronary artery disease	-	-
	Diabetic peripheral neuropathy	-	-
Short stature		+	+
Cardiomyopathy		-	-
Hyperlipidemia		+	-
Developmental delay		-	-
Male pubertal development		-	-
Urologic disease		-	-
Renal disease		-	-
Hepatic disease		-	-
Gastrointestinal disease		-	-
Pulmonary involvement		-	-
Neurologic		-	-

This study was approved by the ethics committee of Children’s Hospital, Fudan University. Informed consent was obtained from the parents, who agreed to join this study, and using the data for scientific research and publication. The methods used in this study were performed in accordance with the approved guidelines.

### Whole genome sequencing

This study was approved by the Ethics Committee of Children’s Hospital, Fudan University. Genomic DNA was sequenced on a HiSeq 2000 sequencer according to the manufacturer’s instructions (Illumina, San Diego, CA, USA). Clean reads were aligned to the reference human genome (UCSC hg19) using a Burrows-Wheeler Aligner (BWA) (v.0.5.9–r16). The average sequencing depth ranged from 35.73X-38.45 X. The mapping rate of clear data ranged from 97.03 to 97.27%, and the genome coverage ranged from 99.83% to 99.85 (Additional file 1: Table S5).

For CNV detection, we employed a MATLAB packet, SegSeq. Only two *de novo* CNVs were identified in the proband. However, both CNVs were reported as polymorphisms in the Database of Genomic Variants (DGV).

**Fig. 2:**
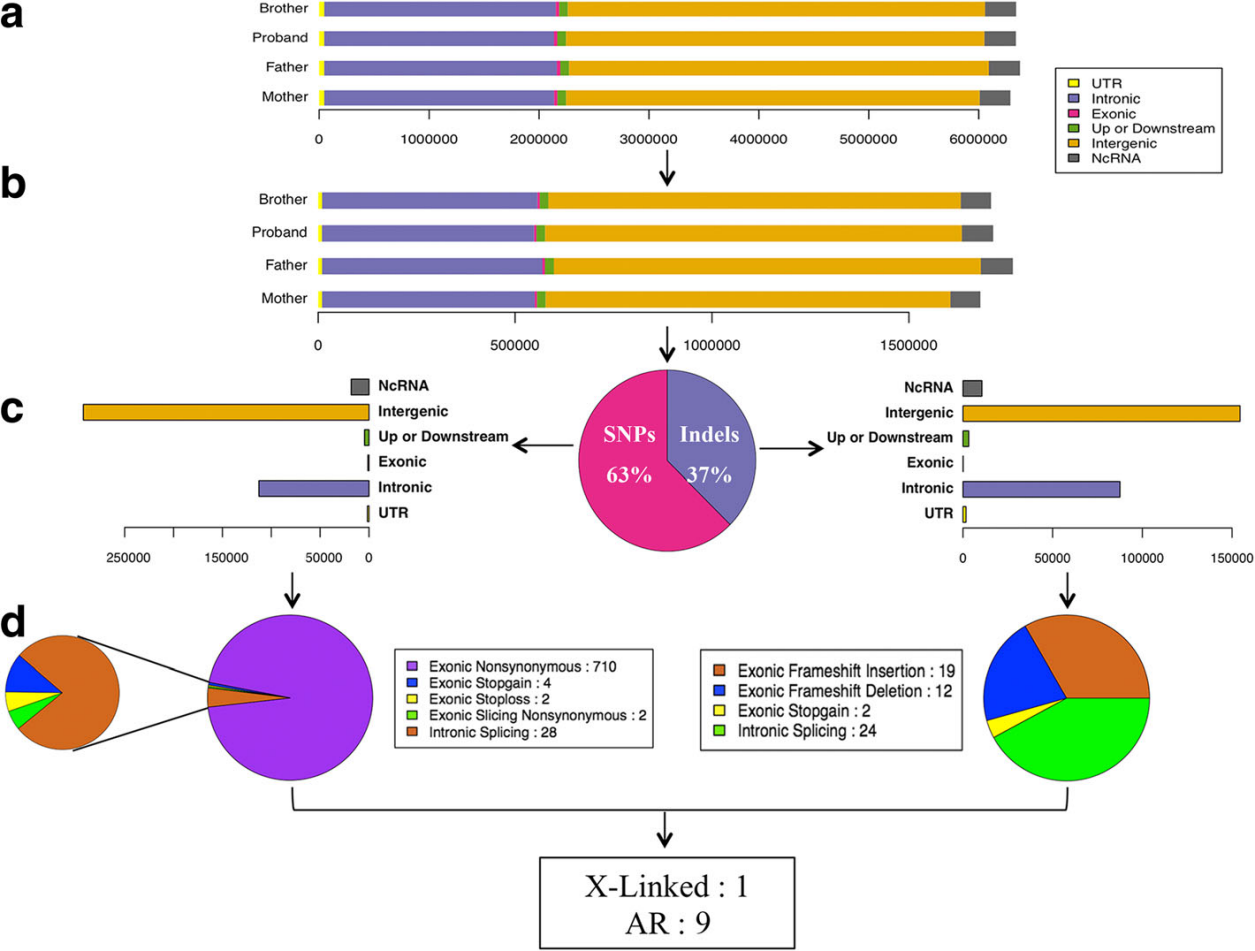
Illustrates the pipeline of whole genome data analysis. **a** The count distribution of variants (SNVs and InDels) from WGS clean data for each individual in the family. The variants were classified into distinct classes according to the genomic regions. **b** The count distribution of variants of unknown significance that do not exist in the dbSNP137 and 1000 Genome Project. **c** the proportion distribution of SNVs and InDels, both of which are shared by two affected siblings. For each group, the variants were divided into different classes according to genomic regions. **d** the distribution of potentially pathogenic mutations classified by mutation functions

Single Nucleotide Variants (SNVs) were detected by SOAPsnp (v.1.05). InDels were identified by GATK. ANNOVAR was used to annotate the variants. The number of variants among samples ranged from 6,288,378 to 6,375,627 (Fig. 2a, Additional file 2: Table S1 and Additional file 3: Table S2). Of these variants, there were 1,681,375 to 1,764,264 potential variants of unknown significance (VUS) that did not exist in the dbSNP137 or 1000 Genome Project (Fig. 2b, Additional file 4: Table S3 and Additional file 5: Table S4). After filtering the known variants from the dbSNP137 and 1000 Genome Project and those without potential function effect, there were 803 candidate pathogenic variants in 399 genes shared between the two affected siblings. The similar phenotypes between the two boys, ALMS negative family history, and absence of consanguinity suggest an X-linked or autosomal recessive inheritance. A total of 10 genes matched the heritance model (Additional file 6: Table S6). Then, we found two heterozygous variants in the *ALMS1* gene (NM_015120.4, .3902C > A, p.S1301X; c.6436C > T, p.R2146X).

Both *ALMS1* mutations were validated by Sanger technique on a 3500XL instrument. S1301X, R2146X are the paternal and maternal variants, respectively. Between the two variants, R2146X was reported most recently in 2015 [9]. S1301X is a novel variant, absent from the Exac, 1000 genome, dbSNP and internal databases: 1554 probands and 1257 families. S1301X, a nonsense mutation located in exon 8, yields a truncated protein missing 2866 amino acids that will likely cause either a partial or complete loss of function. Therefore, we assumed these variants were pathogenic mutations.

Furthermore, different variants between the two siblings were analyzed. A heterozygous missense variant c.1379G > A, p.R460H in *BBS1* gene was only identified in the proband. G at position 1379 is a conserved amino acid. The potential pathogenicity of this variant was scored 0.962, 0.01 and 1 by PolyPhen2, SIFT and MutationTaster, respectively; therefore, the variant was classified as being “damaged”.

## Discussion

It is difficult to definitively diagnose ALMS solely by the clinical features of the two affected siblings. As described by Marshall et al. [2, 10], diagnostic criteria of Alström syndrome have been revised to take the age of the affected person into consideration (Table 1). In our study, the proband with one major (vision) and two minor (diabetes mellitus and hearing) clinical features do not meet the diagnostic criteria for a 15-year-old child. The 8-year-old brother suffering from vision lesion and short stature with just one major and one minor feature, respectively, fails to meet the criteria for a 3- to 14-year-old child. Furthermore, some clinical phenotypes, such as obesity, male hypogonadism, hepatic dysfunction, proteinuria, chronic bronchitis and developmental delay, were reported to occur within the first decade among 50% of Alström patients. However, neither of the affected siblings developed these abnormalities.

The biological function of the ALMS1 protein remains unclear, although localization to centrosomes and basal bodies of ciliated cells suggest apparent roles in ciliary function, intracellular trafficking and adipocyte differentiation [11]. The complex spectrum of traits for ALMS is consistent with those described for ciliopathies, which are genetic disorders resulting from the alterations of various genes. For example,, Bardet-Biedle syndrome (BBS; MIM# 209900) shares many clinical phenotypes with ALMS: retinitis pigmentosa, deafness, and diabetes mellitus [10, 12]. A total of18 genes, excluding *ALMS1*, have been associated with BBS [13]. Previous reports suggested that such interactions might extend to cases of non-Mendelian inheritance in the form of triallelism. This defies the prevailing perception of BBS as an autosomal recessive disease [14–16]. ALMS is a heterogeneous genetic disease with variable expressivity, even within families.

WGS is a powerful tool that provides the full spectrum of genetic variations for such heterogeneous disorders. WGS can not only detect point mutations and small insertions/deletions (InDels) but may also be a useful technique for identifying large copy number variations and the variants in noncoding regions [5, 6].

In our study, we used WGS data filtering (Fig. 2) to discover two compound heterozygous nonsense mutations in *ALMS1* shared by two siblings whose mutations resulted in overlapping clinical presentations between both affected individuals. Thus, we established the diagnosis of ALMS in the two boys.

Previous reports suggested that ciliopathies with a triallelic inheritance defy the prevailing perception among the medical community that BBS is an autosomal recessive disease [17]. For the family in this case study, we identified an extra heterozygous missense variant in the *BBS1* gene in the proband only by WGS and speculate that a *BBS1* gene variant may interact with ALMS1 to alter the onset and course of manifestations of ALMS. More research is required to confirm these results.

## Conclusions

We identified two compound heterozygous *ALMS1* mutations in a Chinese quartet family shared between two siblings suffering cone-rod dystrophy and short stature. We report a novel *ALMS1* mutation and confirmed that WGS is a powerful tool for providing the full spectrum of genetic variations for such heterogeneous disorders.

## Additional files

Additional file 1: Summary of the results of the WGS statistics in the family. (PDF 12 kb)

Additional file 2: Summary of SNV identification in the family. (DOCX 16 kb)

Additional file 3: Summary of InDels identification in the family. (DOCX 16 kb)

Additional file 4: Summary of SNV identification after polymorphism in the dbSNP and 1000 Genome Project were filtered. (DOCX 16 kb)

Additional file 5: Summary of InDels identification after polymorphism in the dbSNP and 1000 Genome Project were filtered. (DOCX 15 kb)

Additional file 6: Summary of rare and inheritance pattern filtered variants identified in the family. (XLSX 12 kb)

## Abbreviations

ALMS: Alström syndrome
CNVs: Copy number variations
DGV: Database of genomic variants
InDels: Insertions/Deletions
NGS: Next-generation sequencing
VUS: Variants of unknown significance
WES: Whole-exome sequencing
WGS: Whole-genome sequencing
