# Supplementary material for: Whole genome sequencing identifies a novel ALMS1 gene mutation in two Chinese siblings with Alström syndrome
Source: BMC Med Genet. 2017 Jul 19;18:75. doi: 10.1186/s12881-017-0418-3 (PMC5518093; doi:10.1186/s12881-017-0418-3)
Supplement: Supplementary file 1 — Summary of the results of the WGS statistics in the family. (PDF 12 kb) [file 12881_2017_418_MOESM1_ESM.pdf]

Additional file 1 Summary of WGS statistics result in the family

WGS statistics	Proband	Brother	Mother	Father
Raw reads	1,329,167,624	1,248,056,386	1,233,150,314	1,272,370,156
Clean reads	1,281,801,928	1,206,649,174	1,191,089,244	1,222,016,642
Clean data(G)	115.36	108.6	107.2	109.98
Sequencing depth	38.45	36.2	35.73	36.66
Reads mapped to genome	1,246,044,401	1,173,677,874	1,160,303,655	1,187,869,090
Map rate(%)	97.03	97.14	97.27	97.07
Duplication rate(%)	6.25	4.66	5.4	4.84
Mean depth of autosomes	36.72	35.28	33.91	35.52
Coverage of genome(%)	99.83	99.83	99.83	99.85
Average read length(bp)	90	90	90	90
Rate of nucleotide mismatch(	0.39	0.34	0.34	0.39
Fraction of genomic covered >=4X(%)	99.58	99.57	99.58	99.59
Fraction of genomic covered >=7X(%)	99.26	99.24	99.25	99.25
Fraction of genomic covered >=10X(%)	98.78	98.69	98.71	98.69
Fraction of genomic covered >=20X(%)	93.63	92.31	92.13	92.43
Mean depth of chrX	18.38	17.59	33.07	17.85
Mean depth of chrY	34.15	33.74	7.3	33.78
Gender test result	M	M	F	M
GC content(%)	41.31	41.46	41.21	41.34
