# Supplementary material for: Whole genome sequencing identifies a novel ALMS1 gene mutation in two Chinese siblings with Alström syndrome
Source: BMC Med Genet. 2017 Jul 19;18:75. doi: 10.1186/s12881-017-0418-3 (PMC5518093; doi:10.1186/s12881-017-0418-3)
Supplement: Supplementary file 2 — Summary of SNV identification in the family. (DOCX 16 kb) [file 12881_2017_418_MOESM2_ESM.docx]

Additional file 2 Summary of SNVs identification in the family

| Item | | Proband | Brother | Mother | Father |
| --- | --- | --- | --- | --- | --- |
| Total | | 5461203 | 5464221 | 5436690 | 5519359 |
| exonic | nonsynonymous SNV | 15446 | 15536 | 15513 | 15871 |
|  | synonymous SNV | 13702 | 13658 | 13742 | 13682 |
|  | stopgain SNV | 209 | 248 | 244 | 255 |
|  | stoploss SNV | 16 | 15 | 15 | 18 |
|  | unknown | 902 | 720 | 767 | 853 |
| exonic;splicing | nonsynonymous SNV | 5 | 7 | 6 | 6 |
|  | synonymous SNV | 5 | 1 | 3 | 4 |
| splicing | | 313 | 314 | 321 | 357 |
| intronic | | 1775320 | 1791633 | 1781871 | 1808480 |
| intergenic | | 3299276 | 3289416 | 3273856 | 3324402 |
| upstream | | 33600 | 33405 | 33596 | 34195 |
| downstream | | 32742 | 32583 | 32729 | 32832 |
| upstream;downstream | | 975 | 1023 | 1011 | 1031 |
| UTR3 | | 31696 | 31970 | 31814 | 31709 |
| UTR5 | | 7404 | 7482 | 7622 | 7509 |
| UTR5;UTR3 | | 15 | 16 | 20 | 13 |
| ncRNA_exonic | | 14529 | 14039 | 14084 | 14685 |
| ncRNA_splicing | | 76 | 86 | 78 | 71 |
| ncRNA_intronic | | 234030 | 231158 | 228468 | 232386 |
| ncRNA_UTR3 | | 795 | 776 | 778 | 825 |
| ncRNA_UTR5 | | 146 | 135 | 151 | 175 |
| ncRNA_UTR5;ncRNA_UTR3 | | 1 | 0 | 1 | 0 |
