# Supplementary material for: Whole genome sequencing identifies a novel ALMS1 gene mutation in two Chinese siblings with Alström syndrome
Source: BMC Med Genet. 2017 Jul 19;18:75. doi: 10.1186/s12881-017-0418-3 (PMC5518093; doi:10.1186/s12881-017-0418-3)
Supplement: Supplementary file 3 — Summary of InDels identification in the family. (DOCX 16 kb) [file 12881_2017_418_MOESM3_ESM.docx]

Additional file 3 Summary of InDels identification in the family

| Item | | Proband | Brother | Mother | Father |
| --- | --- | --- | --- | --- | --- |
| Total | | 877055 | 876217 | 851688 | 856268 |
| exonic | frameshift insertion | 82 | 88 | 82 | 86 |
|  | frameshift deletion | 98 | 100 | 91 | 95 |
|  | nonframeshift insertion | 148 | 143 | 143 | 145 |
|  | nonframeshift deletion | 198 | 193 | 192 | 178 |
|  | stopgain SNV | 4 | 4 | 1 | 4 |
|  | stoploss SNV | 1 | 1 | 1 | 0 |
|  | unknown | 100 | 92 | 89 | 96 |
| exonic;splicing | frameshift deletion | 1 | 1 | 1 | 1 |
|  | nonframeshift insertion | 0 | 0 | 1 | 1 |
| splicing | | 92 | 95 | 94 | 91 |
| intronic | | 313339 | 313752 | 307827 | 307005 |
| intergenic | | 506807 | 505643 | 488253 | 494242 |
| upstream | | 5396 | 5362 | 5298 | 5228 |
| downstream | | 6142 | 6155 | 6006 | 6058 |
| upstream;downstream | | 166 | 171 | 181 | 157 |
| UTR3 | | 6601 | 6665 | 6473 | 6478 |
| UTR5 | | 826 | 809 | 815 | 822 |
| UTR5;UTR3 | | 3 | 4 | 4 | 4 |
| ncRNA_exonic | | 1411 | 1389 | 1380 | 1413 |
| ncRNA_splicing | | 13 | 11 | 12 | 10 |
| ncRNA_intronic | | 35431 | 35361 | 34560 | 33974 |
| ncRNA_UTR3 | | 167 | 153 | 158 | 155 |
| ncRNA_UTR5 | | 28 | 24 | 25 | 24 |
| ncRNA_UTR5;ncRNA_UTR3 | | 1 | 1 | 1 | 1 |
