# Supplementary material for: Whole genome sequencing identifies a novel ALMS1 gene mutation in two Chinese siblings with Alström syndrome
Source: BMC Med Genet. 2017 Jul 19;18:75. doi: 10.1186/s12881-017-0418-3 (PMC5518093; doi:10.1186/s12881-017-0418-3)
Supplement: Supplementary file 4 — Summary of SNV identification after polymorphism in the dbSNP and 1000 Genome Project were filtered. (DOCX 16 kb) [file 12881_2017_418_MOESM4_ESM.docx]

Additional file 4 Summary of SNVs identification after polymorphism in dbSNP and 1000 Genome Porject were filtered

| Item | | Proband | Brother | Mother | Father |
| --- | --- | --- | --- | --- | --- |
| Total | | 1373408 | 1369215 | 1358759 | 1435968 |
| exonic | nonsynonymous SNV | 3687 | 3621 | 3593 | 4088 |
|  | synonymous SNV | 1645 | 1562 | 1614 | 1745 |
|  | stopgain SNV | 116 | 158 | 150 | 175 |
|  | stoploss SNV | 6 | 5 | 5 | 8 |
|  | unknown | 246 | 136 | 150 | 198 |
| exonic;splicing | nonsynonymous SNV | 2 | 3 | 2 | 2 |
|  | synonymous SNV | 2 | 0 | 0 | 1 |
| splicing | | 179 | 186 | 199 | 235 |
| intronic | | 421894 | 430325 | 426507 | 446794 |
| intergenic | | 854583 | 844588 | 838343 | 887525 |
| upstream | | 9544 | 9443 | 9666 | 10148 |
| downstream | | 7973 | 7847 | 7847 | 8294 |
| upstream;downstream | | 247 | 289 | 246 | 268 |
| UTR3 | | 5648 | 5923 | 5782 | 5915 |
| UTR5 | | 1959 | 2022 | 2108 | 2030 |
| UTR5;UTR3 | | 3 | 2 | 4 | 3 |
| ncRNA_exonic | | 3267 | 2926 | 3025 | 3453 |
| ncRNA_splicing | | 23 | 20 | 15 | 17 |
| ncRNA_intronic | | 62142 | 59956 | 59270 | 64808 |
| ncRNA_UTR3 | | 193 | 160 | 182 | 186 |
| ncRNA_UTR5 | | 48 | 43 | 50 | 75 |
| ncRNA_UTR5;ncRNA_UTR3 | | 1 | 0 | 1 | 0 |
