# Supplementary material for: Whole genome sequencing identifies a novel ALMS1 gene mutation in two Chinese siblings with Alström syndrome
Source: BMC Med Genet. 2017 Jul 19;18:75. doi: 10.1186/s12881-017-0418-3 (PMC5518093; doi:10.1186/s12881-017-0418-3)
Supplement: Supplementary file 5 — Summary of InDels identification after polymorphism in the dbSNP and 1000 Genome Project were filtered. (DOCX 15 kb) [file 12881_2017_418_MOESM5_ESM.docx]

Additional file 5 Summary of InDels identification after polymorphism in dbSNP and 1000 Genome Porject were filtered

| Item | | Proband | Brother | Mother | Father |
| --- | --- | --- | --- | --- | --- |
| Total | | 340616 | 339433 | 322616 | 328296 |
| exonic | frameshift insertion | 28 | 34 | 32 | 31 |
|  | frameshift deletion | 25 | 25 | 18 | 29 |
|  | nonframeshift insertion | 39 | 40 | 37 | 42 |
|  | nonframeshift deletion | 54 | 52 | 50 | 45 |
|  | stopgain SNV | 2 | 2 | 0 | 2 |
|  | unknown | 19 | 14 | 11 | 16 |
| splicing | | 30 | 28 | 28 | 26 |
| intronic | | 116527 | 116331 | 113232 | 112316 |
| intergenic | | 203573 | 202568 | 189477 | 196391 |
| upstream | | 2049 | 2025 | 1996 | 1943 |
| downstream | | 2193 | 2184 | 2103 | 2130 |
| upstream;downstream | | 43 | 51 | 54 | 43 |
| UTR3 | | 1988 | 1994 | 1952 | 1908 |
| UTR5 | | 224 | 219 | 217 | 214 |
| UTR5;UTR3 | |  |  |  |  |
| ncRNA_exonic | | 447 | 449 | 445 | 451 |
| ncRNA_splicing | | 4 | 3 | 4 | 3 |
| ncRNA_intronic | | 13312 | 13358 | 12899 | 12648 |
| ncRNA_UTR3 | | 48 | 46 | 51 | 48 |
| ncRNA_UTR5 | | 10 | 9 | 9 | 9 |
| ncRNA_UTR5;ncRNA_UTR3 | | 1 | 1 | 1 | 1 |
